# Molecular characterisation of a rabbit Hepatitis E Virus strain detected in a chronically HEV-infected individual from Germany

**DOI:** 10.1016/j.onehlt.2023.100528

**Published:** 2023-03-22

**Authors:** Patrycja Klink, Dominik Harms, Britta Altmann, Yvonne Dörffel, Ulrike Morgera, Steffen Zander, C. Thomas Bock, Jörg Hofmann

**Affiliations:** a Department of Infectious Diseases, Division of Viral Gastroenteritis and Hepatitis Pathogens and Enteroviruses, Robert Koch Institute, 13353 Berlin, Germany; b Outpatient Clinic, Charité, Universitätsmedizin Berlin, Berlin, Germany; c Institute of Tropical Medicine, University of Tuebingen, Tuebingen, Germany; d Institute of Virology, Charité-Universitätsmedizin Berlin, Freie Universität Berlin, Humboldt-Universität zu Berlin, Berlin Institute of Health, German Centre for Infection Research, Berlin, Germany; e Labor Berlin, Charité-Vivantes GmbH, Berlin, Germany

**Keywords:** Rabbit HEV, Zoonoses, One health, NGS, Resistance-associated substitutions, Ribavirin

## Abstract

In immunocompromised individuals persisting viremia frequently leads to a chronic hepatitis E virus (HEV) infection. Zoonotic transmission of HEV from pigs and wild boar to humans is proven and sporadic infections with rabbit HEV (raHEV) have recently been reported. Here, the molecular characterisation of a raHEV strain isolated from an immunocompromised, chronically HEV-infected, heart-transplanted patient is described. After successful ribavirin (RBV) treatment of a HEV infection in 2019, the patient was again tested HEV positive in 2021 and received a second RBV therapy cycle. Full-length HEV genome amplification and next generation sequencing was performed on a plasma sample taken between first and second cycle of RBV therapy and a stool sample taken two months after starting the second cycle. The sequence of plasma (raHEV-83) and stool (raHEV-99) derived virus showed the highest nucleotide sequence identity to a Chinese raHEV and a phylogenetic relationship to a raHEV strain isolated from a French patient. Furthermore, sequence analysis revealed the presence of RBV-associated substitutions V1479I and G1634K in the HEV sequences from plasma and additionally K1398R from stool. The results underline the role of rabbits as putative sources of HEV infection and emphasize the need of a one health concept for a better understanding of HEV epidemiology and to develop tools for prevention and control of HEV infection.

## Highlights

• Chronic hepatitis E virus (HEV) infection can occur in immunocompromised patients
• A chronically HEV-infected transplant patient who was treated with ribavirin but experienced viral relapse
• Rabbit hepatitis E virus (raHEV) strain was isolated from patients stool and plasma samples identified by NGS
• HEV-sequences showed phylogenetic relationship to Chinese raHEV and mutations associated with antiviral treatment failure
• The finding has one health implication and justifies the need for HEV screening to understand animal-to-human transmission.

## 1 Introduction

Although hepatitis E virus (HEV) is the most common cause of acute viral hepatitis worldwide, its diversity is still not fully known. HEV is the prototype of the family *Hepeviridae* [1]. Human-infecting HEV strains are divided into four genotypes within the *Orthohepevirus A* species, showing distinct epidemiological patterns: HEV-1 and HEV-2 are specific to humans and cause waterborne outbreaks of acute hepatitis mainly on the Asian and African continent. HEV-3 and HEV-4 are transmitted zoonotically and are widespread in industrialised countries. Infections with these genotypes generally take a mild to moderate or asymptomatic course of disease in immunocompetent individuals. HEV replication occurs primarily in the liver and symptoms, if any, resemble those of other forms of viral hepatitis. Extrahepatic manifestations may be caused by neurological, renal, and haematological involvement [2]. However, immunocompromised persons infected with HEV-3 are at higher risk of developing chronic HEV infection, which can further lead to cirrhosis [2,3]. Recently, human HEV infection with HEV-7 related to the consumption of camel meat and milk and infection with rat HEV has been reported [4]. However, zoonotic HEV infection is mainly linked to close contact with or consumption of meat and meat products from domestic pigs and wild boars harbouring HEV-3 or -4 [5,6]. HEV infections are thought to be subclinical in reservoir species such as pigs and wild boar, but transmission rates can be high, as evidenced by high seroprevalence in both domestic pigs, wild boar, and deer [[7], [8], [9]]. In rabbits, HEV was initially detected in 2009 in farmed rabbits in China and subsequently in other countries worldwide, including Germany [[10], [11], [12], [13], [14], [15], [16]]. Experimental infection of rabbits with rabbit HEV (raHEV) resulted in viraemia, faecal viral shedding and elevated liver transaminases with severity of disease depending on the infectious dose [17]. Sporadic cases of human infections with rabbit HEV have been reported from France, Switzerland, Spain, Belgium, and recently from Ireland [12,[18], [19], [20], [21]]. A single report from Germany also observed raHEV in a patient with acute self-limiting hepatitis E [22]. However, the sequence of this strain was not characterised beyond genotyping results, for which the investigated genomic region was not reported. Most of these infections were detected in immunocompromised individuals although the mode of transmission was either unknown or not associated with the consumption of rabbit meat or contact with rabbits. RaHEV strains cluster distinctly from all other known genotypes and are temporarily assigned to HEV-3 (subtype 3-ra) [1]. The raHEV genome consists of three open reading frames (ORFs), but in contrast to any other known HEV genotype most raHEV strains contain a 30–31-amino acid insertion in the X-domain with unclear function [23]. Only few raHEV strains isolated from humans and rabbit do not possess this 3ra-specific insertion [16,21,24].

Chronic HEV infection is usually treated with ribavirin (RBV) and most patients achieve sustained virological response [25]. However, several substitutions in the RNA-dependent RNA-polymerase (RdRp) are associated with treatment failure [26,27]. Individuals infected with HEV-3ra typically respond to RBV treatment [28]. But it is not known to which extent resistance-associated substitutions might impede successful treatment.

In the present study, a raHEV strain was detected in an immunosuppressed, heart-transplanted patient with chronic HEV infection from Germany and the full-length genome sequence was determined using next generation sequencing (NGS). This is the first molecular characterisation of a raHEV strain isolated from a human individual from Germany.

## 2 Materials and methods

### 2.1 Patient sample

In a 59-year old heart-transplanted male patient from Germany, HEV infection was first detected in 2012. In 2019 the patient was again tested positive for HEV after having elevated liver transaminases. Subsequent treatment with RBV for 12 weeks appeared to successfully clear the infection (blood HEV RNA negative by week 8). In April 2021, liver transaminases were again elevated and the patient tested positive for HEV. In May 2021, treatment with 1200 mg RBV was started. Samples collected in April 2021 (plasma; after first RBV cycle) and July 2021 (stool; two months after start of second RBV treatment cycle) were analysed in this study.

### 2.2 Ethics approval and patient consent statement

This study was approved by the local ethics committee (approval number EA1/367/16) and written informed consent was obtained from the patient. Patient samples were de-identified for this study. Patient data, if any, were handled according to the Patient Data Protection Act (PDSG) passed by the Germany's Federal Parliament in 2020 and the General Data Protection Regulation (GDPR / DSGVO) of the European Union. All experiments were performed in accordance with relevant guidelines and regulations.

### 2.3 RNA extraction

RNA was extracted from 200 μl plasma/stool suspension using the Quick-RNA Viral Kit (Zymo Research Europe GmbH, Freiburg, Germany) according to the manufacturer's instruction. RNA was eluted in 25 μl and stored at −80 °C until further use.

### 2.4 Real-time RT-PCR, full-length nested-RT-PCR, and RACE PCR

For quantification, a previously published real-time RT-PCR assay with slight modifications was used [29,30].

The HEV full-length genome was amplified in two overlapping fragments F1 and F2 (expected size of the nested PCR product based on the HEV-3c reference strain FJ705359: F1: 4566 bp; F2: 2642 bp). Two sets of primers were designed (PCR1 and PCR2) based on sequences of HEV subtypes 3c, 3e and 3f, the most common variants in Germany, available at GenBank. Primers used in this study are listed in Table 1.

**Table 1:** Primer sequences.

Primer	Use	Sequence	Location	Source
	Oligo-dT anchor	AAGCAGTGGTATCAACGCAGAGTAC-TTTTTTTTTTTTTTTTTTTTTTTTTTTTTTV		c
	Template switching oligo	GCTAATCATTGCAAGCAGTGGTATCA-ACGCAGAGTACATrGrGrG		c
HEV-F444a	F1-PCR1	CCAYCAGTTYATTAAGGCTCCT	34–55^a^	inhouse
HEV-R39b	F1-PCR1	GCCATRTTCCARACRGTRTTCC	4650–4629^a^	adapted from [30]
HEV-F465	F1-PCR2	GGCTYCTGGCATYACTACTGC	49–69^a^	inhouse
HEV-R27a	F1-PCR2	GGCTCRCCRGARTGYTTCTTCC	4614–4593^a^	adapted from [30]
HEV-F38a	F2-PCR1	GAGGCYATGGTNGAGAARG	4112–4130^a^	adapted from [30]
HEV-R467	F2-PCR1	CCTACCTTCATTTTRAGRCGCTG	7135–7113^a^	inhouse
HEV-F28	F2-PCR2	ATGGAGGAGTGTGGBATGC	4493–4511^a^	adapted from [30]
HEV-R468a	F2-PCR2	CTACCTTCATTYTRAGRCGCTGAAG	7134–7110^a^	inhouse
HEV-479	3′ RACE PCRa (21–0083)	TTG GAT GGG CGG CCG CTT AC	6789–6808^b^	inhouse
cDNA PCR Primer	3′ RACE PCRa +5′ RACE PCRb (21–0083 + 21–0009)	AAG CAG TGG TAT CAA CGC AGA GT		c
TSO Primer	3′RACE PCRb +5′ RACE PCRa (21–0083 + 21–0009)	CAT TGC AAG CAG TGG TAT CAA C		c
HEV-119	5′ RACE PCRa (21–0009)	CGR GGG TAN GGR ACA TAA GGC AT	873–851^b^	inhouse
HEV-480	5′ RACE PCRa (21–0083)	TAG GGC AGA GCG GCG ACA ATT AG	430–408^b^	inhouse
HEV-01	3′RACE PCRa (21–0009)	GTT GTC TCA GCC AAT GGC GAG CC		inhouse
HEV-31a	3′RACE PCRb (21–0009)	CCA GCA GTA YTC YAA RAC ATT YTA TG		inhouse
HEV-478-Mix	5′RACE PCRb (21–0009)	HEV-478-3c - GGC CAG AGG TCA TGC AGA GAR TA HEV-478-3e – GGC CAR AGG TCA TGT AAT GAG TA HEV-478-3f – GGC CAA AGG TCA TGC AGT GAR TA		inhouse
HEV-155	5′RACE sequencing (21–0009)	CGG CCG RAC CAC CAC AGC ATT CGC		inhouse

a Based on HEV-3c reference sequence FJ705359.

b Based on HEV-3ra sequence KX227751.

c NEBNext® Single Cell/Low Input cDNA Synthesis & Amplification Module. PCR1: first-round PCR; PCR2: nested PCR.

For cDNA synthesis, the Maxima reverse transcriptase was used (ThermoFisher Scientific™, Darmstadt, Germany). Reagents mix and cycling protocol are given in Table 2. Clean-up of cDNA was performed using the MagSi-NGS^prep^ Plus Kit (MagnaMedics, Aachen, Deutschland) with elution in 20 μl.

**Table 2:** Reagents and protocol for cDNA synthesis, HEV full-length genome amplification and 3′ and 5′ RACE PCR.

PCR	Reagents (final concentration)	Cycling protocol
cDNA synthesis	5× RT-buffer (1×), dNTP mix (500 μM), TMAC (20 mM), betaine (2 M), ribonuclease inhibitor (20 U), Maxima reverse transcriptase (50 U), oligo dT-anchor (1.25 μM), template switching oligo (3.75 μM) and 4.85 μl of RNA.	45 °C for 45 min, 54 °C for 40 min 58 °C for 20 min, 42 °C for 90 min. Subsequently, 1 μl of RNAse H was added to the mix and incubated at 37 °C for 20 min.
PCR1-F1	5× Q5-buffer (1×), Q5 (0.75 U), dNTPs (200 μM), 5× GC-enhancer (0.5×), HEV-F444a (500 nM), HEV-R39b (800 nM), 2.5 μl of cDNA and water up to a total volume of 25 μl.	98 °C, 30s 40 cycles of: 98 °C for 8 s, 62 °C (∆R 3 °C/s) for 20s, 72 °C for 2 min 50s Final elongation for 5 min at 72 °C
PCR2-F1	5× Q5-buffer (1×), Q5 (0.5 U), dNTPs (200 μM), 5× GC-enhancer (0.5×), HEV-F465 (250 nM), HEV-R27a (400 nM), 0.5 μl PCR1-F1 template and water up to a total volume of 25 μl.	98 °C for 30s 30 cycles of: 98 °C for 8 s, 67 °C (∆R 3 °C/s) for 20s, 72 °C for 2 min 25 s Final elongation for 5 min at 72 °C
PCR1-F2	5× Q5-buffer (1×), Q5 (0.75 U), dNTPs (200 μM), 1,2- propandiole (5%, 0.68 M), TMAC (20 mM), betaine (250 mM), HEV-F38a (800 nM), HEV-R467 (500 nM), 2.5 μl of cDNA and water up to a total volume of 25 μl.	98 °C for 30s 40 cycles of: 98 °C for 8 s, 59 °C (∆R 3 °C/s) for 20s, 72 °C for 1 min 45 s Final elongation for 5 min at 72 °C
PCR2-F2	5× Q5-buffer (1×), Q5 (0.5 U), dNTPs (200 μM), betaine (250 mM), 1,2- propandiole (5%, 0.68 M), HEV-F28 (250 nM), HEV-R468a (400 nM), 0.5 μl of PCR1-F2 template and water up to a total volume of 25 μl.	98 °C for 30s 30 cycles of: 98 °C for 8 s, 66 °C (∆R 3 °C/s) for 20s, 72 °C for 1 min 30s Final elongation for 5 min at 72 °C
3‘RACE PCRa	2× Q5 Master-Mix (1×), Primer-F (500 nM), Primer-R (500 nM), 1 μl cDNA and water up to a total volume of 20 μl.	98 °C for 30s 40 cycles of: 98 °C for 5 s, 61 °C for 20s, 72 °C for 15 s Final elongation for 2 min at 72 °C
3’RACE PCRb	2× Q5 Master-Mix (1×), Primer-F (250 nM), Primer-R (250 nM), 1 μl cDNA and water up to a total volume of 20 μl.	98 °C for 30s 30 cycles of: 98 °C for 5 s, 56 °C for 20s, 72 °C for 15 s Final elongation for 2 min at 72 °C
5‘RACE PCRa	2× Q5 Master-Mix (1×), Primer-F (500 nM), Primer-R (500 nM), 1 μl DNA and water up to a total volume of 20 μl.	98 °C for 30s 10 cycles of: 98 °C for 5 s, 68 °C (∆T − 0.5 °C) for 15 s, 72 °C for 30s 30 cycles of: 98 °C for 5 s, 63 °C for 15 s, 72 °C for 30s Final elongation for 2 min at 72 °C
5‘RACE PCRb	2× Q5 Master-Mix (1×), Primer-F (250 nM), Primer-R (500 nM), 1 μl DNA and water up to a total volume of 20 μl.	98 °C for 30s 6 cycles of: 98 °C for 5 s, 70 °C (∆T − 0.3 °C) for 15 s, 72 °C for 18 s 24 cycles of: 98 °C for 5 s, 68 °C for 15 s, 72 °C for 18 s Final elongation for 2 min at 72 °C

First-round as well as nested PCRs for F1 and F2 were performed using the Q5® High-fidelity PCR Kit (NEB, Frankfurt am Main, Germany) (Table 2). PCR products were analysed on a 1.5% agarose gel.

### 2.5 RACE PCR and Sanger sequencing

3′ and 5′ RACE primers were designed based on the sequences obtained after NGS (Table 2).

RACE PCRs were performed using the Q5 Master mix kit (NEB, Ipswich, USA) on a Biometra Trio cycler (Table 2). PCR products were evaluated on an 1.5% agarose gel and Sanger sequencing performed on an Applied Biosystems 3500 Dx xL sequencer using BigDye™ Terminator v3.1 Cycle Sequencing Kit (Life Technologies, Applied Biosystems, Darmstadt, Germany).

### 2.6 Next generation sequencing

PCR clean-up was performed with Agencourt AMPure XP beads (Beckman Coulter, Pasadena, USA) according to manufacturer's instructions. Purified nested PCR products F1 and F2 from plasma (P—F1 and P—F2) and stool (S—F1 and S—F2) were each pooled at equimolarity. Libraries were prepared using the NEBNext® Ultra™ II FS DNA Library Prep Kit for Illumina and sequenced on an Illumina NextSeq 500 device in a 150 bp paired-end run.

### 2.7 Analysis of raw data

The quality of raw, demultiplexed reads was analysed with fastQC version 0.11.5 [31]. PCR primer sequences were clipped by cutadapt version 1.12 [32]. Primer-clipped reads were presented to a python-based inhouse pipeline which was originally designed for the molecular HIV-1 surveillance [33]. Briefly, adapter sequences were removed using Trimmomatic version 0.36 and all read pairs with an overlap were merged with FLASH version 1.2.11. Trimmomatic was further used for quality trimming. Surviving reads were mapped iteratively to a reference sequence (raHEV strain KX227751, split into two parts analogous to the PCR fragments F1 and F2) with adjusting the reference in each iteration considering potential deletions and insertions using bwa version 0.7.15 [[34], [35], [36]]. The final output was further analysed with CoBaVaDe, a python-based script to generate a consensus sequence with adjustable threshold via codon-based variant detection [33]. Besides a setting for the main viral variant, a Sanger-like threshold of 20% and a threshold of 5% and 1% were applied.

### 2.8 Phylogenetic and recombination analysis

Multiple nucleotide and amino acid sequence alignments with reference sequences according to the latest ICTV recommendation [1] were generated with Geneious Prime 2021.2.2 (http://www.geneious.com/) using MAFFT with default options. 5′ and 3′ sequences generated by RACE-PCR were attached to the consensus sequences obtained after NGS. Gaps in the alignments were stripped and the hypervariable region (HVR) was excluded prior to phylogenetic and recombination analysis. Nucleotide and amino acid sequence similarities to other raHEV strains and HEV genotypes were determined from the % identity matrix implemented in Geneious. Phylogenetic analysis of the full-length genome sequences of the raHEV isolates were performed using IQ-TREE version 1.6.12 [37]. After determining the best fitting model using the substitution model test, a maximum-likelihood phylogenetic tree with 1000 bootstrap values was constructed [38]. Trees were graphically adjusted using iTOL [39].

The full-length sequence of the isolated raHEV strain from plasma was analysed for recombination events using Bootscan supplied by SimPlot version 3.5.1 [40]. Recombination analysis was performed with reference sequences of all HEV genotypes and selected raHEV strains. A sliding window of 700 bp with 30 bp steps was chosen for analysis.

## 3 Results

### 3.1 PCR and NGS

The viral loads in the plasma and stool suspension were 1.44 × 10^6^ and 1.68 × 10^6^ copies/ml, respectively. Fragments F1 and F2 as well as 3′ and 5′ ends were amplified and sequenced successfully for both samples. NGS statistics are summarized in Supplemental Table S1.

After read processing and alignment, consensus sequences of fragments F1 and F2 were merged in Geneious to obtain the full-length sequences. The sequences from the plasma and stool sample are termed raHEV-83 and raHEV-99, respectively, hereafter.

### 3.2 Full-length sequence and comparison of raHEV-83 and raHEV-99 sequences

**Fig. 1:**
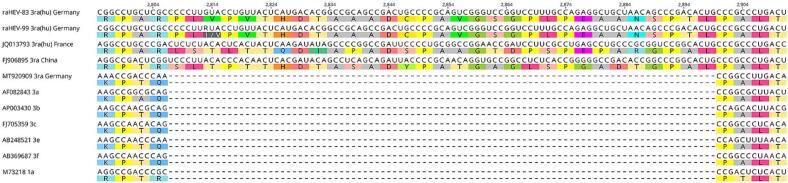
Section of full-length sequence alignment showing positions of the raHEV-specific insertion within the X-domain. The sequences of raHEV-83 and raHEV-99 were aligned to the raHEV strain JQ013793 (isolated from a French patient), the prototypical Chinese raHEV strain FJ906895, the German raHEV strain MT920909 not harbouring the insertion, and selected non-rabbit HEV-1 and -3 reference sequences using MAFFT. Nucleotide positions within the HEV genome given at top. Both nucleotide and amino acid sequences are indicated.

The complete raHEV genome sequences from the plasma (raHEV-83) and stool sample (raHEV-99) consist of 7282 nucleotides, followed by a poly-A tail (accession numbers: OP909735 and OP909736). The 5′ and 3’ UTRs consist of 25 and 71 nucleotides, respectively. Both sequences show the typical organization into three open reading frames (ORF 1: position 26–5194, ORF 2: position 5229–7211 and ORF 3 position 5191–5559) as well as the rabbit-specific 93 nucleotides long insertion in the X-domain (position 2807–2899) (Fig. 1).

Full-length sequences of raHEV-83 and raHEV-99 showed an overall nucleotide sequence identity of 99.5% to each other (corresponding to 17 amino acid changes) using a Sanger-like cut-off of 20%. The viral sequence obtained from the stool sample was more diverse in terms of ambiguities compared to the viral sequence obtained from the plasma sample (0.8% vs. 0.2%, respectively). Comparing the sequences of the main viral variant of raHEV-83 and raHEV-99 revealed a nucleotide sequence identity of 100% to each other and only one amino acid change.

Different cut-offs revealed substitutions in the samples at RdRp positions associated with RBV resistance in HEV-3 infections (Table 3). The substitutions V1479I and G1634K constituted the main viral variant with >98% in both, raHEV-83 and raHEV-99. In raHEV-99, additionally K1383N was detected with 96.3%. Further, the substitutions D1384G, K1383S/N and K1398R were observed in frequencies of <10% or even 5%, respectively in the sequence of raHEV-99.

**Table 3:** RBV resistance-associated substitutions in raHEV-83 and raHEV-99 sequences.

	co20 (%)	co05 (%)	co01 (%)
Plasma (raHEV-83)	V1479I (98.1) G1634K (99.9)		
Stool (raHEV-99)	K1383N (96.3) V1479I (98.9) G1634K (99.9)	D1384G (7.5)	K1383S (3.7) D1384N (4.3) K1398R (1.2)

Substitutions associated with RBV resistance in HEV-3 are underlined. co = cut-off.

### 3.3 Phylogenetic analysis

Phylogenetic analysis of the full-length sequences of raHEV-83 and raHEV-99 was performed together with HEV references sequences as proposed by Smith et al. [1] and all available full-length raHEV sequences from GenBank. A maximum likelihood tree was constructed using IQ-TREE with 1000 bootstrap replicates.

**Fig. 2:**
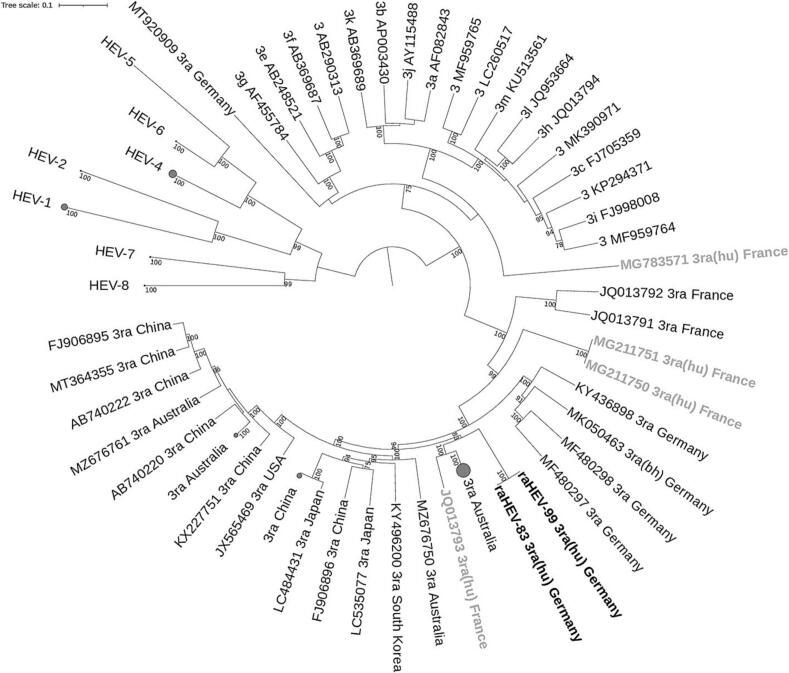
Phylogenetic analysis of raHEV-83 and raHEV-99 full-length sequences. The evolutionary history was inferred by maximum likelihood using the GTR + F + I + G4 model with 1000 bootstrap replicates. Bootstrap values >75% are shown. The tree was rooted at midpoint. Collapsed clades are represented by grey circles with proportional sizing. Sequences from this study are in bold (black). Additionally, raHEV sequences isolated from human hosts are marked in bold (grey). hu: human host, bh: brown hare. (For interpretation of the references to colour in this figure legend, the reader is referred to the web version of this article.)

The phylogenetic analysis shows clustering of the raHEV-83 and raHEV-99 sequences with other raHEV strains and revealed a close relationship to a raHEV strain isolated from a French patient (Acc.No. JQ013793) and various strains from Australia (Fig. 2). Other raHEV strains isolated from rabbits and a brown hare in Germany distinctly clustered in a single clade. Maximum likelihood trees for nucleotide sequences of ORF1, ORF2, ORF3, the single domains within ORF 1 (Met, Y, X, PCP, Hel, RdRp) as well as amino acid sequences of ORF1, ORF2 and ORF3 were constructed. In all modes, the nucleotide sequences of raHEV-83 and raHEV-99 clustered with other raHEV strains, but always distinctly from raHEV strains isolated from German rabbits (data not shown). This was also the case for the amino acid sequences of ORF1 and ORF2 (Supplemental fig. S1). However, the ORF3 amino acid sequences showed raHEV-83 and raHEV-99 at the basal position of all other raHEV sequences.

On the nucleotide level, full-length sequences of raHEV-83 and raHEV-99 showed the highest identity (85.4%) to a raHEV strain from China (Acc.No. KX227751) (Supplemental table S2). Nucleotide sequence identity to other raHEV strains was between 77.0% to 85.1% with an identity between 76.9% and 84.4% to raHEV strains from Germany. The maximal nucleotide sequence identity to other HEV-3 strains was 79.7%, while it was between 74.3% to 75.7% to HEV of other genotypes. Nucleotide sequence as well as amino acid identity was highest to other raHEV strains in all regions of the genome.

### 3.4 Recombination analysis

**Fig. 3:**
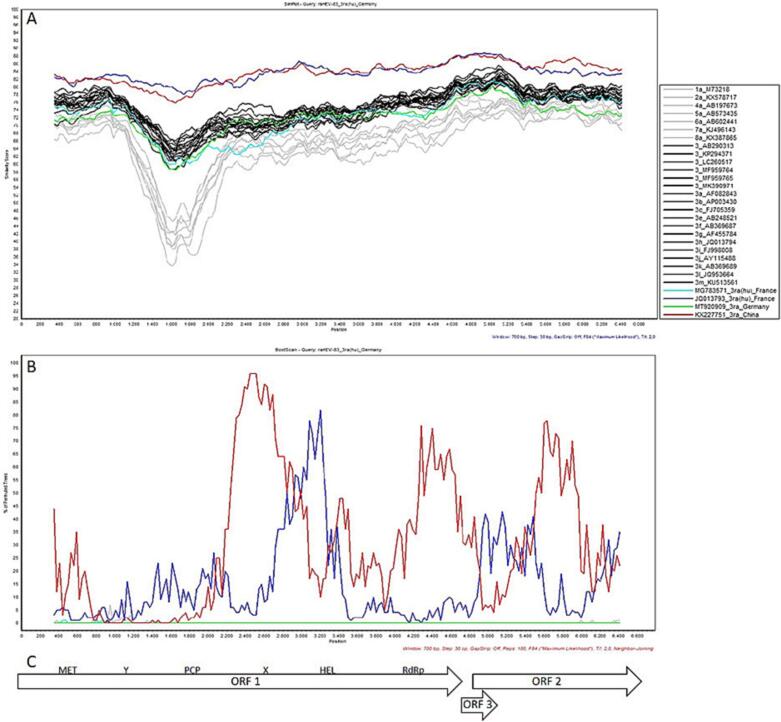
Recombination analysis of raHEV-83 full-length sequence. A) SimPlot analysis with similarity score in %. B) Bootscan analysis in % of permuted trees. C) Schematic representation of the HEV genome organization.

For recombination analysis (Fig. 3), only the sequence of raHEV-83 was analysed due to the high degree of similarity to raHEV-99. SimPlot analysis showed that along the whole genome sequence raHEV-83 has the highest similarity to other typical raHEV strains and only a low sequence similarity to the recently identified recombinant raHEV strain from a French individual (Acc.No. MG783571) and a raHEV strain of a putative novel HEV-3 subtype isolated from a German rabbit (Acc.No. MT920909). The sequence similarity was low to other HEV-3 subtypes and all other HEV genotypes.

No evidence for intra-genotypic recombination was observed. However, recombination with a Chinese raHEV strain (Acc.No. KX227751) is highly supported, especially in the X-domain of ORF1 (96% of permuted trees) and with lower support values also in the RdRp domain and ORF2. This is supported by the high nucleotide sequence identity of KX227751 to the sequence of raHEV-83. In the helicase domain, recombination with the French raHEV strain isolated from a human individual (Acc.No. JQ013793) was evident, although with a maximal support value of only 82%.

## 4 Discussion

In this study, a rabbit hepatitis E virus (raHEV) has been isolated and characterised from a heart-transplanted patient from Germany. The patient tested positive for HEV in 2012 and 2019 and seemingly cleared the infection under RBV treatment in 2019. However, in 2021, viral recurrence was observed. Subsequently, raHEV was detected in a plasma (raHEV-83; after first and before second RBV treatment cycle) and stool sample (raHEV-99; two months after starting second cycle).

Phylogenetic analyses of raHEV-83 and raHEV-99 revealed high sequence identity rates to raHEV strains isolated from rabbits and human. Although close genetic relationship between raHEV strains from rabbits and humans has been shown in past studies, until now, infections of human individuals with raHEV could not be traced back to the contact to rabbits or consumption of meat of these animals [21,28]. The patient in our study stated to have had contact with his neighbour's rabbits between 2012 and 2019. Unfortunately, no samples are available to verify a zoonotic transmission. Likewise, differentiation between viral reactivation and reinfection in 2019 and 2021 cannot be made. Nonetheless. our finding emphasizes the contribution of rabbits to human HEV infection and for the first time, temporal relationship between infection with a HEV-3ra strain and direct contact with rabbits could be established.

A number of amino acid substitutions in the RdRp are associated with RBV treatment failure in chronic HEV-3 infections [27,41]. The V1479I substitution was detected at a frequency of 98.1% after the first RBV treatment cycle in the plasma sample. Additionally, a G1634K substitution was detected at a frequency of 99.9%. Compared to human HEV-3 strains, both substitutions are highly prevalent in HEV-3ra isolates. Nicot and colleagues analysed 16 raHEV strains and detected V1479I in 100% and G1634K in 56% of the sequences [42]. The combination of V1479I and G1634K has also been detected in 100% of sequences from individuals acutely infected with HEV-1 from Bangladesh [43]. It has been shown that a similar substitution, G1634R, is associated with treatment failure in HEV-3 infections in vivo [26]. However, it is not known, whether V1479I or G1634K confer similar effects in HEV-3ra-infected individuals. The patient in this study received RBV after HEV was detected in 2019. Unfortunately, no resistance analysis was performed and it is not possible to reconstruct if the substitutions V1479I and G1634K were already present within the viral quasispecies during or even before the first treatment. Two months after starting the second RBV treatment cycle, HEV was still detectable and further RBV-associated substitutions emerged in the main viral variant (K1383N), but also in low-level frequency viral variants (D1384G and K1398R) isolated from the patient's stool sample. This suggests a selection of these additional variants early in the second cycle. Previous studies observed a selection of these substitutions during RBV therapy in patients failing to achieve sustained virological response [26,27]. It is not known to which extent these substitutions also confer resistance in HEV-3ra infections. Selection of resistance-associated substitution in the main but also low-level frequency viral variants reported in this study, as well as reported prolonged shedding of HEV in faeces during RBV therapy, emphasize at the risk for RBV treatment failure in HEV-3ra-infected patients. In addition, the presence of other yet uncharacterized polymorphisms in raHEV may confer resistance to a certain degree.

Our study is limited by the lack of sequence analysis prior to the first RBV treatment in 2019. Therefore, it cannot be determined whether the V1479I and G1634K mutations were selected by the first RBV cycle or already present in the baseline main variants. Although direct transmission of HEV from the pet rabbits cannot be proven for certain, a human raHEV infection after close contact to pet rabbits is reported for the first time in this study.

## 5 Conclusion

In this study, a raHEV strain has been detected in a chronically HEV-infected heart transplanted patient from Germany and the genome molecularly characterised. Although infections with raHEV in humans are only rarely reported, they underline the need for a one health concept to better understand the epidemiology of HEV in humans and animals and therefore support prevention and control of HEV infections. Our study underlines the relevance of detecting patient-specific viral variants that may adversely impact treatment outcome using sensitive methods. The applied methods and results of this study therefore have major implications for future one health, diagnostic, prognostic, and personalized medicine concepts.

## Funding

This study was supported by a grant from the German 10.13039/501100003107Federal Ministry of Health (BMG) regarding a decision of the German Bundestag by the Federal Government (NiCaDe-Project Phase I and II, grant No: ZMVI-2519GHP711, and CHED-Project grant No: ZMVI1-2518FSB705). D.H. is supported by the 10.13039/100007570Claussen-Simon-Stiftung (10.13039/100007570Claussen-Simon Foundation; CSF) “Dissertation Plus” program, Germany, and the Fazit-Stiftung “Promotions Stipendium”. B.A. is supported by ProFIT grant of the Investitionsbank Berlin, Germany (IBB, ProFIT No. 10169028 / co-funded by EFRE (No. 10169096)). The funders BMG, CSF, Fazit, and ProFit had no role in study design, data collection and interpretation, or the decision to submit the work for publication. The content is the responsibility only of the authors and does not represent the views of the funders.

## CRediT authorship contribution statement

**Patrycja Klink:** Conceptualization, Data curation, Formal analysis, Investigation, Methodology, Resources, Software, Validation, Visualization, Writing – original draft, Writing – review & editing. **Dominik Harms:** Formal analysis, Software, Validation, Visualization, Writing – original draft, Writing – review & editing. **Britta Altmann:** Formal analysis, Investigation, Methodology, Software, Validation, Writing – review & editing. **Yvonne Dörffel:** Resources, Writing – review & editing. **Ulrike Morgera:** Resources, Writing – review & editing. **Steffen Zander:** Formal analysis, Investigation, Writing – review & editing. **C. Thomas Bock:** Conceptualization, Methodology, Project administration, Resources, Supervision, Validation, Funding acquisition, Writing - review & editing. **Jörg Hofmann:** Conceptualization, Methodology, Project administration, Resources, Supervision, Validation, Writing – review & editing.

## Declaration of Competing Interest

The authors declare that they have no known competing financial interest and personal relationship that could inappropriately influence the presented work.

## Data Availability

The displayed data are sufficient to draw the conclusions presented in this manuscript. Further data can be made available upon reasonable request to the first or corresponding author. The sequences generated in this study are available at the National Center for Biotechnology Information (NCBI) GenBank under the accession numbers listed in the Material and Methods section (OP909735 and OP909736).

## Appendix A Supplementary data

Supplementary material Image 1
