# Supplementary material for: Molecular characterisation of a rabbit Hepatitis E Virus strain detected in a chronically HEV-infected individual from Germany
Source: One Health. 2023 Mar 22;16:100528. doi: 10.1016/j.onehlt.2023.100528 (PMC10288053; doi:10.1016/j.onehlt.2023.100528)
Supplement: Supplementary file 1 — Supplementary material [file mmc1.pdf]

Supplementary material

Molecular characterisation of a rabbit HEV strain detected in a chronically HEV-infected individual from Germany

Patrycja Klink [a], Dominik Harms [a, 1], Britta Altmann [a], Yvonne Dörffel [b], Ulrike Morgera [b], Steffen Zander [a], C.-Thomas Bock [a, c, §, *], Jörg Hofmann [d, e, §]

[a] Department of Infectious Diseases, Division of Viral Gastroenteritis and Hepatitis Pathogens and Enteroviruses, Robert Koch Institute, 13353 Berlin, Germany

[b] Outpatient Clinic, Charité, Universitätsmedizin Berlin, Berlin, Germany

[c] Institute of Tropical Medicine, University of Tuebingen, Tuebingen, Germany

[d] Institute of Virology, Charité-Universitätsmedizin Berlin, corporate member of Freie Universität Berlin, Humboldt-Universität zu Berlin, Berlin Institute of Health, and German Centre for Infection Research, Berlin, Germany

[e] Labor Berlin, Charité-Vivantes GmbH, Berlin, Germany

[1] Present address (D.H.): Institute of Cardiac Diagnostics and Therapy, Berlin, Germany

[§] C.-T.B. and J.H. contributed equally to this work and thus shared last authorship.

* **Corresponding author at**: Robert Koch Institute, Seestrasse 10, D-13353 Berlin, Germany; Tel.: +49 (0) 30 18754 2379; E-mail address: BockC@rki.de (C.-Th. Bock)

Table S1

NGS statistics.

	raHEV-83	raHEV-99
raw reads	2x 3590128	2x 5902116
merged pairs	2285116 (63.65%)	2991192 (50.68%)
trimmed reads	4199451	6993505
length after trimming (mean)	153.84	156.71
remained unmapped	21492 (0.5 %)	135184 (1.9 %)
mapping score (mean)	149.23 (F1); 149.75 (F2)	148.66 (F1); 150.96 (F2)
read depth (mean)	80478.39 (F1); 92228.40 (F2)	136015.74 (F1); 124036.13 (F2)

Table S2

Nucleotide sequence (nt) and amino acid (aa) identity matrix of raHEV-83.

**nt identity (in %)**	**HEV-1**	**HEV-2**	**HEV-3**	**HEV 3ra (Acc.No.)**	**HEV-4**	**HEV-5**	**HEV-6**	**HEV-7**	**HEV-8**
Full-length	74.6	74.8	79.7	**85.4** (KX227751)	75.8	74.8	74.2	75.7	75.6
ORF1	72.8	73.1	78.8	**84.6** (JQ013793)	73.9	72.8	72.8	74.5	74.4
ORF2	79.2	78.8	83.4	**87.9** (AB740221, LC484431)	80.8	79.8	78	78.6	79.6
ORF3	82.7	83	89.1	**93.6** (LC484431, MZ676770)	84.2	82.7	82	84.8	81.5
Met	76.3	76.9	81.9	**84.7** (KX227751)	76.8	75.8	76.3	75.6	78
Y	79.3	78.8	83.0	**85.9** (MZ676770)	79.9	79.1	79.8	78.6	78.8
PCP	64.8	64.8	73.8	**82.2** (JX565469, MZ676765)	63.5	60.7	62.1	65.6	64.8
X	73.3	74.8	81.5	**86.6** (MF480297)	76.2	75.8	73.6	76	76.6
Hel	74.8	73.8	78.8	**82.6** (MZ676765, MZ676766)	76.5	75.8	73.1	74.3	74.6
RdRp	75.2	74.1	80.9	**86.9** (JQ013793)	75.8	72.3	74.9	77.6	76.2
**aa identity (in %)**	**HEV-1**	**HEV-2**	**HEV-3**	**HEV 3ra**	**HEV-4**	**HEV-5**	**HEV-6**	**HEV-7**	**HEV-8**
ORF1	84.5	84.9	91.1	**94.8**	86.7	84.5	84	87.3	85.9
ORF2	90.1	89.7	94.2	**95.9**	91.2	89.6	88.5	89.4	89.9
ORF3	79.4	80.4	88.8	**92.5**	84.1	75.7	77.6	78.5	76.6

MET: methyltransferase, Y: Y-domain, PCP: papain-like cysteine protease, X: X-domain, HEL: helicase, RdRp: RNA-dependent RNA-polymerase, Acc.No: Accession number. Accession numbers for aa identity are not listed in the table, due to multiple sequences with similar identity level. Highest identities are marked in bold.

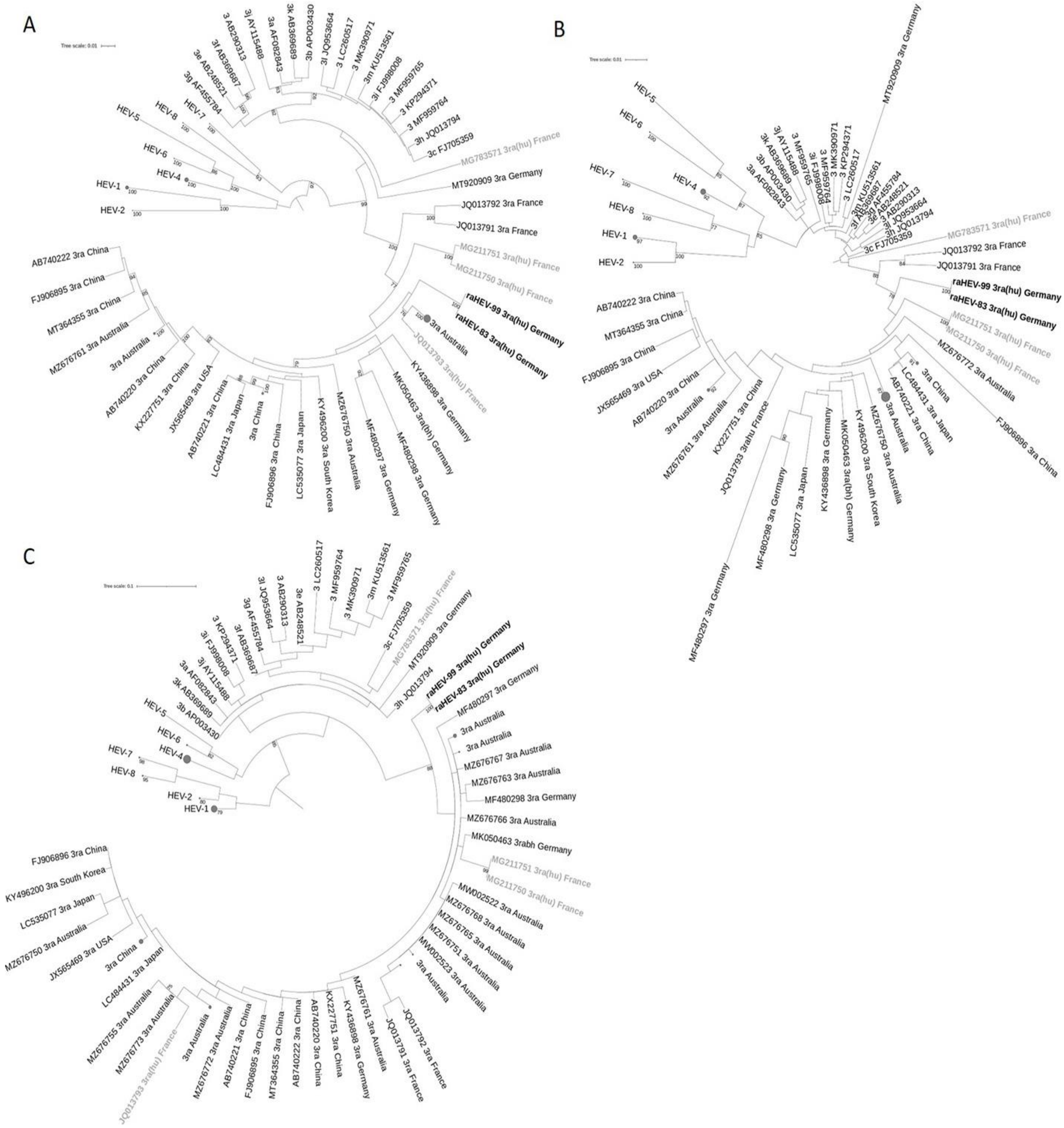

Figure S1: Phylogenetic analysis of raHEV-83 and raHEV-99 ORF 1 (**A**), ORF 2 (**B**) and ORF 3 (**C**) amino acid sequences. The phylogenetic tree was constructed by maximum likelihood using the JTT model with 1000 bootstrap replicates. Bootstrap values >75% are shown. The tree was rooted at midpoint. Collapsed clades are represented by grey circles with proportional sizing. Sequences from this study are in bold (black). Additionally, raHEV sequences isolated from human hosts are marked in bold (grey). Hu: human host, bh: brown hare.
